# Hyperspectral Image Classification with Spatial Filtering and *ℓ*_2,1_ Norm

**DOI:** 10.3390/s17020314

**Published:** 2017-02-08

**Authors:** Hao Li, Chang Li, Cong Zhang, Zhe Liu, Chengyin Liu

**Affiliations:** 1 School of Mathematics and Computer Science, Wuhan Polytechnic University, Wuhan 430023, China; lihao@whpu.edu.cn (H.L.); hb_wh_zc@163.com (C.Z.); 2 School of Electronic Information and Communications, Huazhong University of Science and Technology, Wuhan 430074, China; zheliu@hust.edu.cn (Z.L.); liuchengyin@hust.edu.cn (C.L.)

**Keywords:** alternating direction method of multipliers, hyperspectral classification, outliers, spatial filtering and *ℓ*_2,1_ norm (SFL)

## Abstract

Recently, the sparse representation based classification methods have received particular attention in the classification of hyperspectral imagery. However, current sparse representation based classification models have not considered all the test pixels simultaneously. In this paper, we propose a hyperspectral classification method with spatial filtering and $\ell_{2,1}$ norm (SFL) that can deal with all the test pixels simultaneously. The $\ell_{2,1}$ norm regularization is used to extract relevant training samples among the whole training data set with joint sparsity. In addition, the $\ell_{2,1}$ norm loss function is adopted to make it robust for samples that deviate significantly from the rest of the samples. Moreover, to take the spatial information into consideration, a spatial filtering step is implemented where all the training and testing samples are spatially averaged with its nearest neighbors. Furthermore, the non-negative constraint is added to the sparse representation matrix motivated by hyperspectral unmixing. Finally, the alternating direction method of multipliers is used to solve SFL. Experiments on real hyperspectral images demonstrate that the proposed SFL method can obtain better classification performance than some other popular classifiers.

## 1. Introduction

Over the past few decades, hyperspectral imagery has been widely used in different remote sensing applications owing to its high-resolution spectral information of the materials in the scene [1,2,3]. Various hyperspectral image classification techniques have been presented for a lot of real applications including material recognition, urban mapping and so on [4,5,6,7,8].

To date, a lot of hyperspectral image classification methods have been presented. Among them, the most representative method is the support vector machine (SVM) [9], which has shown desirable hyperspectral image classification performance. Recently, the sparse representation based classification methods have received a lot of attention in the area of image analysis [10,11,12,13,14], particularly in the classification of hyperspectral image. Chen et al. introduced a dictionary-based sparse representation framework for hyperspectral classification [15]. To be specific, a test pixel is sparsely represented by a few labeled training samples, and the class is determined as the one with the minimal class-specific representation error. In addition, Chen et al. also proposed the simultaneous orthogonal match pursuit (SOMP) to utilize the spatial information of hyperspectral data [15]. To take the additional structured sparsity priors into consideration, Sun et al. reviewed and compared several structured priors for sparse representation based hyperspectral image classification [16], which can exploit both the spatial dependences between the neighboring pixels and the inherent structure of the dictionary. In [17], Chen et al. extended the joint sparse representation to the kernel version for hyperspectral image classification, which can provide a higher classification accuracy than the conventional linear sparse representation algorithms. In addition, Liu et al. proposed a class-specific sparse multiple kernel learning framework for hyperspectral image classification [18], which determined the associated weights of optimal base kernels for any two classes and led to better classification performances. To take other spectral properties and higher order context information into consideration, Wang et al. proposed the spatial-spectral derivative-aided kernel joint sparse representation for hyperspectral image classification [19], and the derivative-aided spectral information can complement traditional spectral features without inducing the curse of dimensionality and ignoring discriminating features. Moreover, Li et al. proposed the joint robust sparse representation classification (JRSRC) method to take the sparse representation residuals into consideration, which can deal with outliers in hyperspectral classification [20]. To integrate the sophisticated prior knowledge about the spatial nature of the image, Roscher et al. proposed constructing a novel dictionary for sparse-representation-based classification [21], which can combine the characteristic spatial patterns and spectral information to improve the classification performance. In order to adaptively explore the spatial information for different types of spatial structures, Fu et al. proposed a new shape-adaptive joint sparse representation method for hyperspectral image classification [22], which can construct a shape-adaptive local smooth region for each test pixel. In order to capture the class-discriminative information, He et al. proposed a group-based sparse and low-rank representation to improve the dictionary for hyperspectral image classification [23]. To take different types of features into consideration, Zhang et al. proposed an alternative joint sparse representation by the multitask joint sparse representation model [24]. To overcome the high coherence of the training samples, Bian et al. proposed a novel multi-layer spatial-spectral sparse representation framework for hyperspectral image classification [25]. In addition, to take the class structure of hyperspectral image data into consideration, Shao et al. proposed a probabilistic class structure regularized sparse representation method to incorporate the class structure information into the sparse representation model [26].

It had been argued in [27] that the collaborative representation classification can obtain very competitive classification performance, while the time consumption was much lower than that of sparse representation. Thus, various collaborative representation methods had been proposed for hyperspectral image classification. Li et al. proposed the nearest regularized subspace (NRS) classifier by using the distance-weighted Tikhonov regularization [28]. Then, the Gabor filtering based nearest regularized subspace classifier had been proposed to exploit the benefits of using spatial features [29]. Collaborative representation with Tikhonov regularization (CRT) had also been proposed for hyperspectral classification [30]. The main difference between NRS and CRT was that the NRS only used within-class training data for collaborative representation while the latter adopted all the training data simultaneously [30]. In [31], the kernel version of a collaborative representation was proposed and denoted as kernel collaborative representation classifier (KCRC). In addition, Li et al. proposed proposed combining the sparse representation and collaborative representation for hyperspectral image classification to make a balance between sparse representation and collaborative representation in the residual domain [32]. Moreover, Sun et al. combined the active learning and semi-supervised learning to improve the classification performance when given a few initial labeled samples, and proposed the extended random walker [33] algorithm for the classification of hyperspectral image.

Very recently, some deep models had been proposed for hyperspectral image classification [34]. To the best of our knowledge, Chen et al. proposed a deep learning method named stacked autoencoder for hyperspectral image classification in 2014 [35]. Recently, convolutional neural networks have been very popular in pattern recognition, computer vision and remote sensing. Convolutional neural networks usually contained a number of convolutional layers and a classification layer, which can learn deep features from the training data and exploit spatial dependence among them. Krizhevsky et al. trained a large convolutional neural networks to classify the 1.2 million high-resolution images in the ImageNet, which had obtained superior image classification accuracy [36]. Since then, convolutional neural networks had been applied for hyperspectral image classification [37,38], which had achieved desirable classification performance. To take the spatial information into consideration, a novel convolutional neural networks framework for hyperspectral image classification using both spectral and spatial features was presented [39]. In addition, Aptoula et al. proposed a combined strategy of both attribute profiles and convolutional neural networks for hyperspectral image classification [40]. To overcome the imbalance between dimensionality and the number of available training samples, Ghamisi et al. proposed a self-improving band selection based convolutional neural networks method for hyperspectral image classification [41]. In addition, some patch based convolutional neural networks hyperspectral image classification methods had also been proposed, such as the method in [42,43]. In order to achieve low computational cost and good generalization performance, Li et al. proposed combining convolutional neural networks with extreme learning machines for hyperspectral image classification [44]. Furthermore, Shi et al. proposed a 3D convolutional neural networks (3D-CNN) method for hyperspectral image classification that can take both the spectral and spatial information into consideration [45].

However, all of the above mentioned methods, whether they are based on sparse representation, collaborative representation or deep models, adopt the pixel-wise classification strategy, i.e., they do not consider all the pixels simultaneously. In [46], theoretical work has demonstrated that multichannel joint sparse recovery is superior to applying standard sparse reconstruction methods to each single channel individually, and the probability of recovery failure decays exponentially with the increase in the number of channels. In addition, the probability bounds still hold true even for a small number of signals. For the classification of hyperspectral images, the multichannel means recovering multi hyperspectral pixels simultaneously. Therefore, inspired by the theoretical work in [46], in this paper, we propose a hyperspectral classification method with spatial filtering and $\ell_{2,1}$ norm (SFL) to deal with all the test samples simultaneously, which can not only take much less time but also obtain comparable good or better classification performance. First, the $\ell_{2,1}$ norm regularization is adopted to select correlated training samples among the whole training data set. Meanwhile, the $\ell_{2,1}$ norm loss function which is robust for outliers is also implemented. Second, we adopt the simple strategy in [47] to exploit the local continuity, and all the training and testing samples are spatially averaged with their nearest neighbors to take the spatial information into consideration, which can be seen as spatial filtering. Third, the non-negative constraint is added in the sparse representation coefficient matrix motivated by hyperspectral unmixing. Finally, to solve SFL, we use the alternating direction method of multipliers [48], a simple but powerful algorithm that is well suited to distributed convex optimization.

The main contribution of this work lies in proposing an SFL for hyperspectral classification that can deal with all the test pixels simultaneously. Experiments on real hyperspectral images demonstrate that the proposed SFL method can obtain better classification performance than some other popular classifiers.

## 2. Related Work

In this section, we briefly introduce the classical sparse representation for the classification of hyperspectral images, which can be found in [16]. It is assumed that the pixels in the same class lie in the same low-dimensional subspace, and it has *K* different classes. Therefore, for an unknown test sample $y\in R^{B}$, where *B* denotes the the number of bands, y is assumed to lie in the union of the *K* different subspaces, which can seen as the sparse linear combination of all the training samples

(1) $$\begin{array}{cc} y & =A^{1}x^{1}+A^{2}x^{2}+\cdots +A^{K}x^{K}\\ & =\left[A^{1}\cdots A^{K}\right]\left[\begin{array}{c} x^{1}\\ \vdots \\ x^{K} \end{array}\right]=Ax. \end{array}$$

Therefore, given the dictionary of training samples $A\in R^{B\times M}$, where *M* is the number of training samples. For an unknown test sample y, the sparse representation coefficient vector $x\in R^{M}$ can be obtained by solving the optimization problem as follows:

(2) $$\hat{x}=\arg\;\min_{x}{\;∥y-Ax∥}_{2}^{2}+\lambda {∥x∥}_{1},$$

where A consists of the class subdictionaries ${\left\{A^{k}\right\}}_{k=1,\cdots ,K}$, and *λ* is the regularization parameter. In addition, Equation (2) can be solved by the alternating direction method of multipliers in [49]. Thus, the class label of x is determined as the one with the minimal class-specific reconstruction residual:

(3) $$\mathrm{Class}\left(y\right)=\arg\;\min_{k=1,\cdots ,K}{∥y-A^{k}{\hat{x}}^{k}∥}_{2}^{2}.$$

## 3. Proposed Classifiers

In [46], it has been proved that, with the increase in the number of channels, the failure probability of sparse reconstruction decreases exponentially. Thus, multichannel sparse reconstruction is superior to single channel sparse reconstruction. In addition, the probability bounds are valid even for a small number of signals. Based on this theory, we deal with all the test samples simultaneously, and the proposed SFL classification method will be briefly described.

Let $Y=\left[y_{1},\;y_{2},\cdots ,y_{N}\right]\in R^{B\times N}$, where ${\left\{y_{n}\right\}}_{n=1,\cdots ,N}$ denotes the columns of Y, and *N* denotes the number of test pixels. To deal with all the test pixels simultaneously, it is natural that the sparse representation coefficient matrix $X=\left[x_{1},\;x_{2},\cdots ,x_{N}\right]\in R^{M\times N}$ for all the test pixels can be obtained by solving the optimization problem as follows:

(4) $$\hat{X}=\arg\;\min_{X}{\;∥Y-AX∥}_{F}^{2}+\lambda {∥X∥}_{1},$$

which also can be solved by the alternating direction method of multipliers in [49]. ${∥.∥}_{F}$ represents the matrix Frobenius norm, which is equal to the Euclidean norm of the vector of singular values, i.e.,

(5) $${∥X∥}_{F}=\sqrt{\langle X,X\rangle }={\left(\sum_{i=1}^{M}\sum_{j=1}^{N}X_{ij}^{2}\right)}^{\frac{1}{2}}={\left(\sum_{i=1}^{r}\sigma_{i}^{2}\right)}^{\frac{1}{2}},$$

where $\sigma_{i}$ (i=1,...,r) denotes the singular value of X. After the optimized $\hat{X}$ is obtained, the classes of all test pixels can be obtained by the minimum class reconstruction error:

(6) $$\mathrm{Class}\left(y_{n}\right)=\arg\;\min_{k=1,\cdots ,K}{∥y_{n}-A^{k}{\hat{x_{n}}}^{k}∥}_{2}^{2},\;n=1,\cdots ,N.$$

However, Equation (4) adopts the pixel-wise independent regression, which ignores the correlation among the whole training data set. Recent research shows that the high-dimensional data space is smooth and locally linear, and it has been versified in image reconstruction and classification problems [50,51]. For joint consideration of the classification of neighborhoods, in this paper, we introduce the $\ell_{2,1}$ norm regularization and adapt it to extract correlated training samples among the whole training data set with joint sparsity, which is defined as follows:

(7) $${∥X∥}_{2,1}=\sum_{i=1}^{M}\sqrt{\sum_{j=1}^{N}X_{ij}^{2}}.$$

The $\ell_{2,1}$ norm was first introduced by Ding et al. [52], which makes the traditional principal component analysis more robust for outliers. The outliers are defined as data points that deviate significantly from the rest of data. Traditional principal component analysis optimizes the sum of squared errors, since the few data points that have large squared errors will dominate the sum. Therefore, the traditional principal component analysis is sensitive to outliers. It has been shown that minimizing the $\ell_{1}$ norm is more robust and can resist a larger proportion of outliers compared with quadratic $\ell_{2}$ norms [53]. The $\ell_{2,1}$ norm is identical to a rotational invariant $\ell_{1}$ norm, and the solution of $\ell_{2,1}$ norm based robust principal component analysis is the principal eigenvectors of a more robust re-weighted covariance matrix, which can alleviate the effects of outliers. In addition, the $\ell_{2,1}$ norm has the advantage of being rotation invariant compared with the $\ell_{1}$ norm [52,54,55], i.e., applying the same rotation to all points has no effect on its performance. Due to the above-mentioned advantages, the $\ell_{2,1}$ norm has been applied in feature selection [56], multi-task learning [57], multi-kernel learning [58], and non-negative matrix factorization [59]. Nie et al. [56] introduced the $\ell_{2,1}$ norm to feature selection, and they used $\ell_{2,1}$ norm regularization to select features across all data points with joint sparsity. The $\ell_{2,1}$ norm based loss function is used to remove outliers, and the feature selection process is proved to be effective and efficient.

Similarly, we adopt the $\ell_{2,1}$ norm regularization to select correlated training samples among the whole training data set with joint sparsity for hyperspectral image classification. Thus, the corresponding optimization problem is as follows:

(8) $$\hat{X}=\arg\;\min_{X}{\;∥Y-AX∥}_{F}^{2}+\lambda {∥X∥}_{2,1},$$

which can be solved by the alternating direction method of multipliers in [60]. This model can be seen as an instance of the methodology in [61], which can impose sparsity across the pixels both at the group and individual levels. In addition, to make it more robust for outliers, the $\ell_{2,1}$ norm loss function is adopted. Thus, the corresponding optimization problem is as follows:

(9) $$\hat{X}=\arg\;\min_{X}{\;∥Y-AX∥}_{2,1}+\lambda {∥X∥}_{2,1}.$$

Due to limited resolution of hyperspectral image sensors and the complexity of ground materials, mixed pixels can easily be found in hyperspectral images. Therefore, a hyperspectral unmixing step is needed [62,63]. Hyperspectral unmixing is a process to identify the pure constituent materials (endmembers) and estimate the proportion of each material (abundance) [64]. The linear mixture model has been prevalently used in hyperspectral unmixing, and the abundance is considered to be non-negative in a linear mixture model [65]. If we deem A as the spectral library consisting of endmembers, then X can be seen as the abundance matrix. Therefore, X is also non-negative. When adding the non-negative constraint into the sparse representation matrix, the corresponding optimization problem is as follows:

(10) $$\hat{X}=\arg\;\min_{X\geq 0}{\;∥Y-AX∥}_{F}^{2}+\lambda {∥X∥}_{2,1},$$

(11) $$\hat{X}=\arg\;\min_{X\geq 0}{\;∥Y-AX∥}_{2,1}+\lambda {∥X∥}_{2,1}.$$

In addition, since the spectral signatures of neighboring pixels are highly correlated, which make them belong to the same material with high probability, we thus adopt the simple strategy in [47] to exploit the local continuity, and all the training and testing samples are spatially averaged with their nearest neighbors to take the spatial information into consideration, which can be seen as spatial filtering. Moreover, when *N*=1, it is easy to see that Equation (8) reduces to Equation (2), and Equation (9) reduces to the optimization problem as follows:

(12) $$\hat{x}=\arg\;\min_{x}{\;∥y-Ax∥}_{1}+\lambda {∥x∥}_{1}.$$

**Figure 1:**
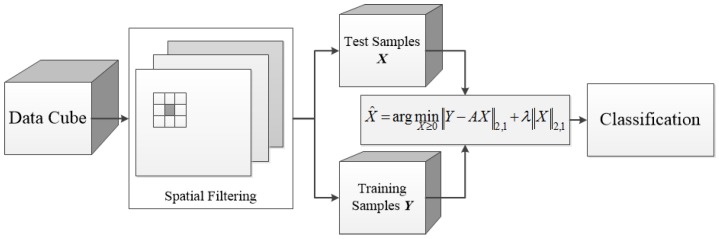
Flow chart of the proposed method.

To sum up, the detailed procedure of our proposed method can be seen from Figure 1. Finally, to solve the optimization problem from Equation (9) to Equation (12), Equation (10) can be solved by the alternating direction method of multipliers in [60], and Equations (9) and (12) are special cases of Equation (11). Thus, it comes down to solving Equation (11). For simplification, Equation (11) can be written as:

(13) $$\min_{X}{\;∥AX-Y∥}_{2,1}+\lambda {∥X∥}_{2,1}+l_{R_{+}}\left(X\right),$$

where $l_{R_{+}}\left(X\right)=\sum_{i=1}^{P}l_{R_{+}}\left(X_{i}\right)$ is the indicator function of nonnegative quadrant $R_{+}$, and $X_{i}$ is the *i*-th column of X. If $X_{i}$ belongs to the nonnegative quadrant, then $l_{R_{+}}\left(X_{i}\right)$ is zero. If not, it is +∞.

In order to solve Equation (11), the alternating direction method of multipliers [48] method is implemented. By introducing auxiliary variables P, Q and W, Equation (11) could be rewritten as:

(14) $$\begin{array}{cc} \min_{X} & {\;∥P∥}_{2,1}+\lambda {∥W∥}_{2,1}+l_{R_{+}}\left(X\right),\\ s.t. & \;AQ-Y=P,\\ & \;Q=W,\\ & \;Q=A. \end{array}$$

A compact version of it is:

(15) $$\min_{V,Q}\;g\left(V\right)\;s.t.\;GQ+BV=Z,$$

where $g\left(V\right)={∥P∥}_{2,1}+\lambda {∥W∥}_{2,1}+l_{R_{+}}\left(A\right)$, $G=\left[\begin{array}{c} A\\ I\\ I \end{array}\right]$, $B=\left[\begin{array}{ccc} -I & 0 & 0\\ 0 & -I & 0\\ 0 & 0 & -I \end{array}\right]$, $Z=\left[\begin{array}{c} Y\\ 0\\ 0 \end{array}\right]$, V≡(P,W,X), and I is the unit matrix. Thus, the augmented Lagrangian function could be expressed as:

(16) $$L\left(V,Q,\Lambda \right)=g\left(V\right)+\frac{\mu }{2}{∥GQ+BV-Z-\Lambda ∥}_{F}^{2},$$

where μ>0, Λ/μ stands for the Lagrange multipliers. In order to update P, we solve

(17) $$P^{k+1}=\arg\;\min_{P}{\;∥P∥}_{2,1}+\frac{\mu }{2}{∥AQ^{k}-Y-P-\Lambda_{1}^{k}∥}_{F}^{2},$$

and its solution is the famous vector soft threshold operator [10], which updates each row independently

(18) $$P^{k+1}\left(r,:\right)=\text{vect-soft}\left(\zeta \left(r,:\right),\frac{1}{\mu }\right),$$

where $\zeta =AQ^{k}-Y-\Lambda_{1}^{k}$, and the vect-soft-threshold function $g\left(b,\tau \right)=b\frac{\max\{∥b{∥}_{2}-\tau ,0\}}{\max\{∥b{∥}_{2}-\tau ,0\}+\tau }$. To update W, we solve

(19) $$\begin{array}{c} W^{k+1}=\arg\;\min_{W}{\;\lambda ∥W∥}_{2,1}+\frac{\mu }{2}{∥Q^{k}-W-\Lambda_{2}^{k}∥}_{F}^{2}, \end{array}$$

and its solution is also the vector soft threshold operator [10]:

(20) $$W^{k+1}\left(r,:\right)=\text{vect-soft}\left(\gamma \left(r,:\right),\frac{\lambda }{\mu }\right),$$

where $\gamma =Q^{k}-\Lambda_{2}^{k}$.

To update X, we solve

(21) $$\begin{array}{cc} X^{k+1} & =\arg\;\min_{X}\;l_{R_{+}}\left(X\right)+\frac{\mu }{2}{∥Q^{k}-X-\Lambda_{3}^{k}∥}_{F}^{2}\\ & =\max\;(Q^{k}-\Lambda_{3}^{k},0). \end{array}$$

To update Q, we solve

(22) $$\begin{array}{cc} Q^{k+1}= & \arg\;\min_{Q}\;{∥AQ-Y-P^{k+1}-\Lambda_{1}^{k}∥}_{F}^{2}+\\ & ∥Q-W^{k+1}-\Lambda_{2}^{k}{∥}_{F}^{2}+{∥Q-X^{k+1}-\Lambda_{3}^{k}∥}_{F}^{2},\\ = & {\left(A^{T}A+2I\right)}^{-1}[A^{T}\left(Y+P^{k+1}+\Lambda_{1}^{k}\right)+W^{k+1}+\\ & \Lambda_{2}^{k}+X^{k+1}+\Lambda_{3}^{k}]. \end{array}$$

The stopping criterion is $∥GQ^{k}+BV^{k}{-Z∥}_{F}^{2}<\varepsilon *\sqrt{\left(J*K\right)}$, where *ε* is the error threshold, and *J* and *K* are the number of rows and columns of Z. *μ* is updated in the same way as [48], which keeps the ratio between the alternating direction method of multiplier primal norms and dual residual norms within a given positive interval. Based on this, we can get Proposition 1, whose proof of convergence is given in [48].

**Proposition** **1.** *Function g in Equation* (15) is closed, proper, and convex. If there exist solutions $V^{*}$ and $Q^{*}$, then iterative sequence $\left\{V^{k}\right\}$ and $\left\{Q^{k}\right\}$ converge to $V^{*}$ and $Q^{*}$, respectively. If not, at least one of $\left\{V^{k}\right\}$ and $\left\{Q^{k}\right\}$ diverge [48].

## 4. Experiments

### 4.1. Experimental Data

**Figure 2:**
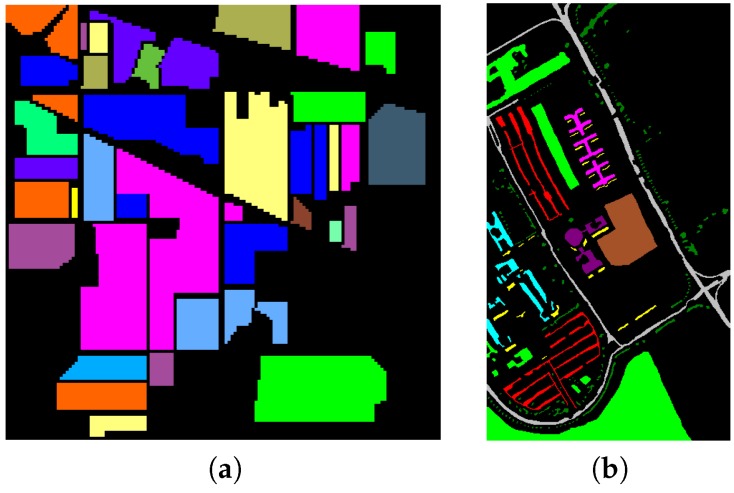
Ground truth image of (**a**) Indian Pines; (**b**) Pavia University.

Two datasets are used in the experiment. The first dataset is Indiana Pines obtained by Airborne Visible/Infrared Imaging Spectrometer (AVIRIS) in 1992. The image size is 145 × 145, and 220 bands are taken in the spectral range from 0.4–2.5 μm. After removal of water absorption bands (No. 104–108, 150–163, 220), 200 bands are used, and the ground truth image is shown in Figure 2a. There are 16 material classes in Indiana Pines and 10,249 labeled samples. In addition, 1027 samples (about 10%) are used as training data, as shown in Table 1. Thus, the rest is used for testing.

The second dataset is Pavia University obtained by a Reflective Optics System Imaging Spectrometer (ROSIS) in 2001 at Paiva University, Pavia, Italy. The size of the image is 610 × 340 with a spatial resolution of 1.3 m. The number of bands is 103, and the ground truth image is shown in Figure 2b. There are nine classes and 42,776 labeled samples, 426 of them (about 1%) are chosen as the training data, and the others are used as test data, as shown in Table 2.

### 4.2. Parameter Setting

In experiments, we mainly compare the classification performance when using the pixel-wise strategy and dealing with all the test pixels simultaneously. In addition, we also made a step-by-step comparison by adding or removing spatial filtering and/or constraints to see which step’s contribution is more important. For these methods, there are mainly five parameters: i.e., the neighbor size *T*, the regularization parameter *λ*, the Lagrange multiplier regularization parameter *μ*, the error tolerance *ε* and the maximum number of iteration. The neighbor size *T* and the regularization parameter *λ* play an important role in the proposed method, which control the size of spatial filtering and the trade-off between fidelity to the data and sparsity of the solution, respectively. While the Lagrange multiplier regularization parameter *μ*, the error tolerance *ε* and the maximum number of iteration, which have lesser impact on the efficiency of the corresponding algorithms, are set to a fixed value, i.e., $\mu =10^{-2}$, $\varepsilon =10^{-6}$, and the maximum number of iteration is 1000. For the neighbor size *T*, we use the same parameter setting in [16]. For the Indian Pine data set, a spatial window of 9×9 (*T* = 81) is adopted, which is due to this image consisting of mostly large homogeneous regions. For the University of Pavia data set, a spatial window of 5×5 (T = 25) is used, which is due many narrow regions being present in this image. The regularization parameter *λ* is chosen from the given intervals {$10^{-6}$, $10^{-5}$, $10^{-4}$, $10^{-3}$, $10^{-2}$, $10^{-1}$}.

**Figure 3:**
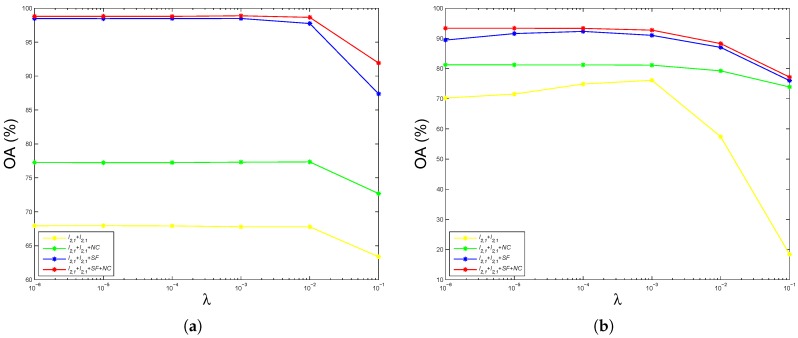
Performance of overall accuracy as a function of the parameter *λ* using the hyperspectral image of (**a**) Indian Pines; (**b**) Pavia University.

Figure 3 shows the performance of overall accuracy as a function of the regularization parameter *λ* using the hyperspectral image of Indian Pines and Pavia University. For convenience, the “Spatial Filtering” and “Non-negative Constraint” are abbreviated as “SF” and “NC”, respectively. For example, for the “$\ell_{2,1}$+$\ell_{2,1}$+SF+NC”, the first “$\ell_{2,1}$” denotes the loss function norm, the second “$\ell_{2,1}$” denotes the regularization term norm, “SF” denotes using the spatial filtering, and “NC” denotes using the non-negative constraint. Thus, they are the same as the abbreviation of the other compared methods. It can be seen from Figure 3 that the overall accuracy remains stable when $\varepsilon <10^{-2}$. It then decreases when $\varepsilon >10^{-2}$. In addition, “$\ell_{2,1}$+$\ell_{2,1}$+SF+NC” and “$\ell_{2,1}$+$\ell_{2,1}$+SF” have much better overall accuracy than “$\ell_{2,1}$+$\ell_{2,1}$+NC” and “$\ell_{2,1}$+$\ell_{2,1}$”, respectively, which demonstrate that it is significant to improve the overall accuracy when taking the spatial filtering into consideration. Moreover, “$\ell_{2,1}$+$\ell_{2,1}$+SF+NC” and “$\ell_{2,1}$+$\ell_{2,1}$+NC” have better overall accuracy than “$\ell_{2,1}$+$\ell_{2,1}$+SF” and “$\ell_{2,1}$+$\ell_{2,1}$”, respectively, which demonstrate that it helps to improve the overall accuracy when taking the non-negative constraint into consideration. Furthermore, the elevation of overall accuracy when using the spatial filtering is much larger than those when using the non-negative constraint, which suggests that the spatial filtering has a larger effect on the overall accuracy than the non-negative constraint.

### 4.3. Classification Performance

**Figure 4:**
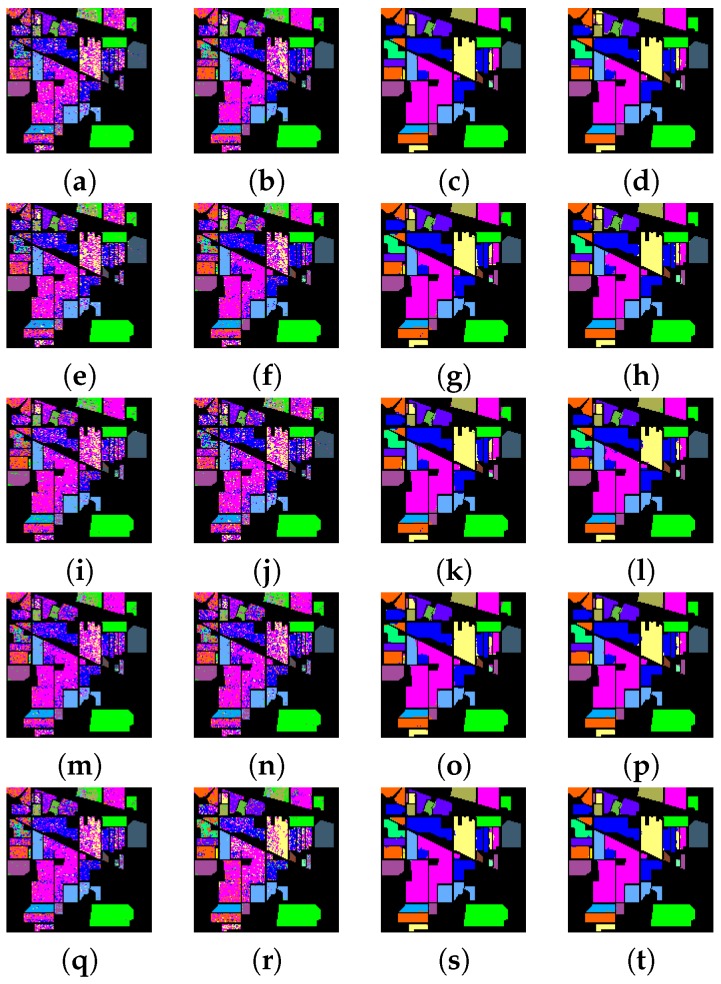
Classification maps for the Indian Pines data set. (**a**) $\ell_{2}$+$\ell_{1}$; (**b**) $\ell_{2}$+$\ell_{1}$+NC; (**c**) $\ell_{2}$+$\ell_{1}$+SF; (**d**) $\ell_{2}$+$\ell_{1}$+SF+NC; (**e**) $\ell_{1}$+$\ell_{1}$; (**f**) $\ell_{1}$+$\ell_{1}$+NC; (**g**) $\ell_{1}$+$\ell_{1}$+SF; (**h**) $\ell_{1}$+$\ell_{1}$+SF+NC; (**i**) *F*+$\ell_{1}$; (**j**) *F*+$\ell_{1}$+NC; (**k**) *F*+$\ell_{1}$+SF; (**l**) *F*+$\ell_{1}$+SF+NC; (**m**) *F*+$\ell_{2,1}$; (**n**) *F*+$\ell_{2,1}$+NC; (**o**) *F*+$\ell_{2,1}$+SF; (**p**) *F*+$\ell_{2,1}$+SF+NC; (**q**) $\ell_{2,1}$+$\ell_{2,1}$; (**r**) $\ell_{2,1}$+$\ell_{2,1}$+NC; (**s**) $\ell_{2,1}$+$\ell_{2,1}$+SF; (**t**) $\ell_{2,1}$+$\ell_{2,1}$+SF+NC (SFL).

**Figure 5:**
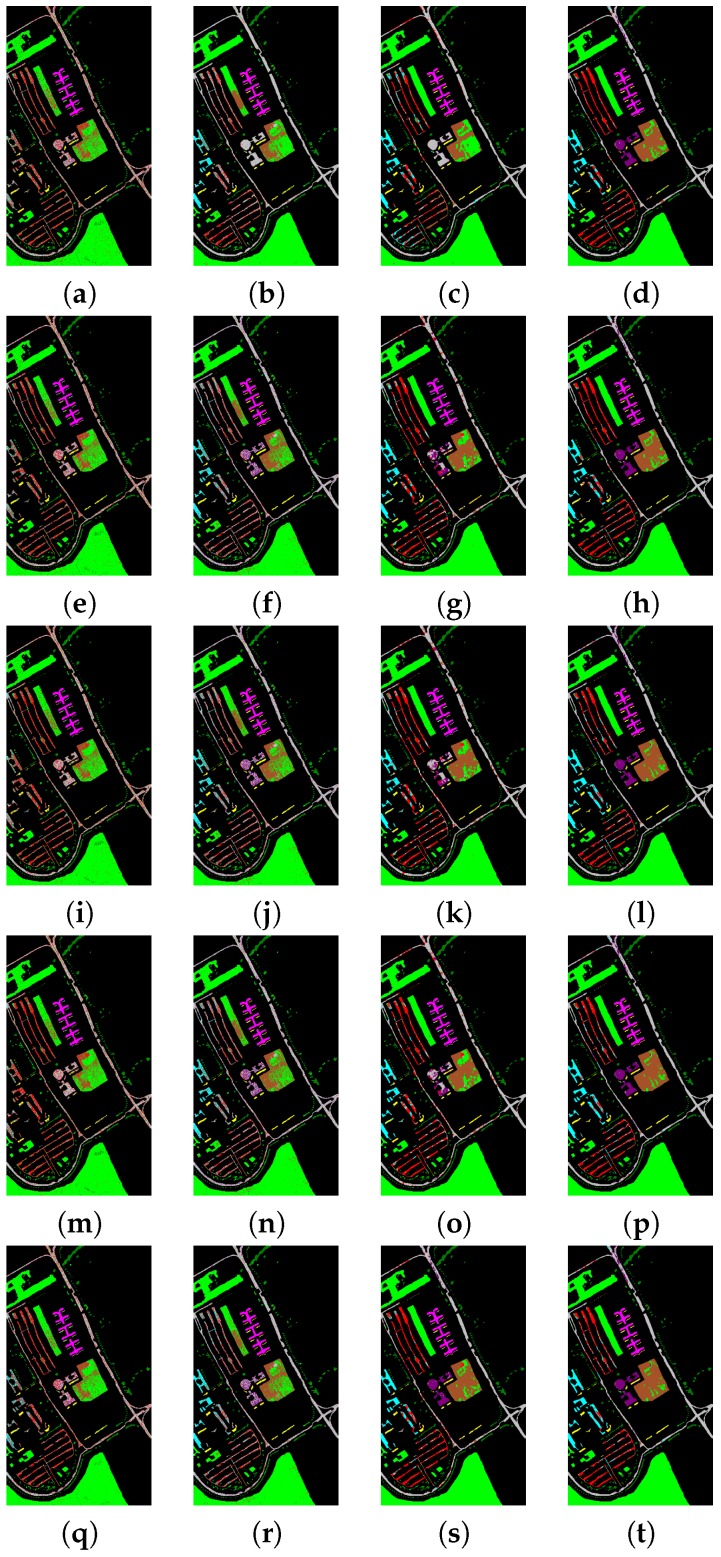
Classification maps for the Pavia University data set. (**a**) $\ell_{2}$+$\ell_{1}$; (**b**) $\ell_{2}$+$\ell_{1}$+NC; (**c**) $\ell_{2}$+$\ell_{1}$+SF; (**d**) $\ell_{2}$+$\ell_{1}$+SF+NC; (**e**) $\ell_{1}$+$\ell_{1}$; (**f**) $\ell_{1}$+$\ell_{1}$+NC; (**g**) $\ell_{1}$+$\ell_{1}$+SF; (**h**) $\ell_{1}$+$\ell_{1}$+SF+NC; (**i**) *F*+$\ell_{1}$; (**j**) *F*+$\ell_{1}$+NC; (**k**) *F*+$\ell_{1}$+SF; (**l**) *F*+$\ell_{1}$+SF+NC; (**m**) *F*+$\ell_{2,1}$; (**n**) *F*+$\ell_{2,1}$+NC; (**o**) *F*+$\ell_{2,1}$+SF; (**p**) *F*+$\ell_{2,1}$+SF+NC; (**q**) $\ell_{2,1}$+$\ell_{2,1}$; (**r**) $\ell_{2,1}$+$\ell_{2,1}$+NC; (**s**) $\ell_{2,1}$+$\ell_{2,1}$+SF; (**t**) $\ell_{2,1}$+$\ell_{2,1}$+SF+NC (SFL).

The experiments are performed on a desktop with 3.5 GHz Intel Core CPU, 64 GB memory and Matlab Code. To evaluate the classification performance of different methods, the overall accuracy, average accuracy and kappa statistic [16] are used to evaluate the performances of these methods. Table 3 and Table 4 show the classification performances for Indian Pines data set when using the pixel-wise strategy and dealing with all the test pixels simultaneously, respectively. It can be seen from Table 3 and Table 4 that methods using the spatial filtering generally obtain better overall accuracy, average accuracy and kappa statistics than those without spatial filtering. For example, “$\ell_{2}$+$\ell_{1}$+SF+NC” and “$\ell_{2}$+$\ell_{1}$+SF” have much better overall accuracy than “$\ell_{2}$+$\ell_{1}$+NC” and “$\ell_{2}$+$\ell_{1}$”, respectively, which demonstrates that it helps a lot to improve overall accuracy by using the spatial filtering. In addition, methods using the non-negative constraint generally obtain better overall accuracy than those without non-negative constraints. For example, “$\ell_{1}$+$\ell_{1}$+SF+NC” and “$\ell_{1}$+$\ell_{1}$+NC” have better overall accuracy than “$\ell_{1}$+$\ell_{1}$+SF” and “$\ell_{1}$+$\ell_{1}$”, respectively, which demonstrates that it helps to improve overall accuracy by using the non-negative constraint. It also can be clearly seen that the spatial filtering has a larger effect on the classification performance than the non-negative constraint. Moreover, methods using $\ell_{2,1}$ norm regularization term can generally obtain better classification performance than methods using $\ell_{1}$ norm regularization term, for example, “*F*+$\ell_{2,1}$+SF+NC” and “*F*+$\ell_{2,1}$” generally have better overall accuracy than “*F*+$\ell_{1}$+SF+NC” and “*F*+$\ell_{1}$”, respectively, which demonstrate that it is beneficial to select correlated training samples among the whole training data set, and can impose sparsity across the pixels both at the group and individual levels. Furthermore, methods using $\ell_{2,1}$ norm loss function can generally obtain better classification performance than methods using *F* norm loss function. For example, “$\ell_{2,1}$+$\ell_{2,1}$+SF+NC” and “$\ell_{2,1}$+$\ell_{2,1}$” generally have better overall accuracy than “*F*+$\ell_{2,1}$+SF+NC” and “*F*+$\ell_{2,1}$”, respectively, which demonstrate that the $\ell_{2,1}$ norm loss function is more robust for outliers than *F* norm loss function. Table 5 and Table 6 show the classification performances for Pavia University data set when using the pixel-wise strategy and dealing with all the test pixels simultaneously, respectively. We can also obtain the above-mentioned conclusion from Table 5 and Table 6 when using the Pavia University data. In addition, from Table 3, Table 4, Table 5 and Table 6, it can be observed that these methods when dealing with all the test pixels simultaneously can obtain comparable or better overall accuracy than these regression based pixel-wise sparse representation methods, and they are much faster than these pixel-wise sparse representation methods, which demonstrates that it is significant to considerer all the test pixels simultaneously. Figure 4 and Figure 5 show the classification maps for the Indian Pines and Pavia University data sets, respectively, which can give a visual comparison between different methods.

**Figure 6:**
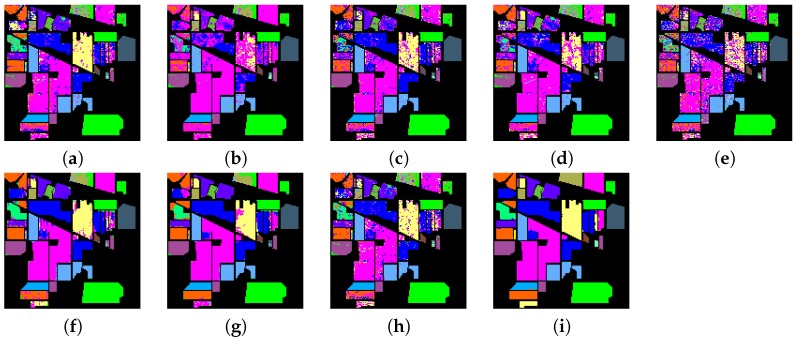
Classification maps for the Indian Pines data set using the compared methods and the proposed method. (**a**) SVM; (**b**) NRS; (**c**) CRT; (**d**) KCRC; (**e**) OMP; (**f**) SOMP; (**g**) JRSRC; (**h**) 3D-CNN; (**i**) SFL.

**Figure 7:**
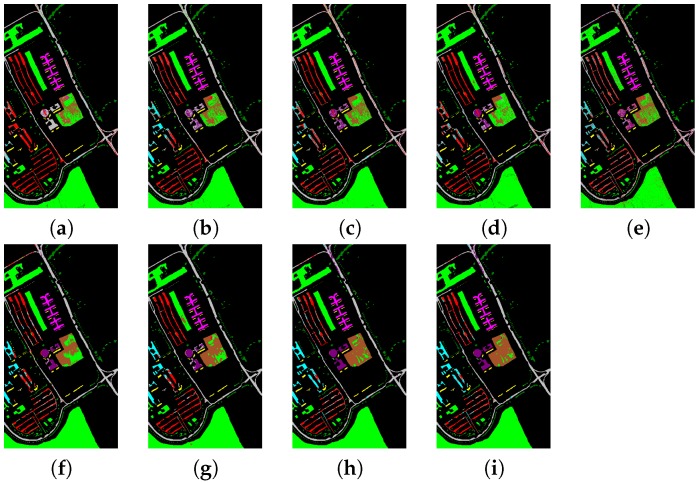
Classification maps for the Pavia University data set using the compared methods and the proposed method. (**a**) SVM; (**b**) NRS; (**c**) CRT; (**d**) KCRC; (**e**) OMP; (**f**) SOMP; (**g**) JRSRC; (**h**) 3D-CNN; (**i**) SFL.

We also choose other eight methods for comparison, i.e., SVM [9,66], NRS [28,67], CRT [30,67], KCRC [31,68], OMP [15], SOMP [15], JRSRC [20] and 3D-CNN [45,69]. The SVM is a very popular classifier, the 3D-CNN is a deep neural network based classifier, and the other six compared methods are collaborative representation and sparse representation based classifiers. Table 7 and Table 8 show the classification performances of the proposed SFL and eight compared methods using the Indian Pines and Pavia University data set, respectively. In addition, Figure 6 and Figure 7 show the classification maps of the Indian Pines and Pavia University data set when using the proposed SFL and eight compared methods, which can give a visual comparison between different methods. From Table 7 and Table 8, it can be clearly seen that the proposed SFL can obtain the best classification performance, which demonstrates that our proposed SFL is efficient for hyperspectral image classification. In addition, the SVM is the fastest, the reason lies in that it is implemeted in C Lagnuage which is much faster than Matlab. NRS, CRT and KCRC are very fast due to the fact that they are collaborative representation methods, and they have closed solutions, which do not need iteration. The OMP and SOMP are also very fast due to the fact that they are greedy sparse representation methods, while the JRSRC method is very time-consuming due to the fact that JRSRC is a regression based sparse representation method. In addition, the 3D-CNN is not fast because the main time-consuming aspect lies in the training. Our proposed method is also a regression based method, which takes more time than the collaborative representation methods and greedy sparse representation methods. There are several possible ways for us to improve the time consumed in the process. One way is to use C Language and graphic processing unit for fast implementation. Another way is to use the first-order primal-dual algorithm in [70] to achieve faster convergence.

## 5. Conclusions

In this paper, we propose an SFL method for a hyperspectral image classification method based on the multichannel joint sparse recovery theory in [46], which can deal with all the test pixels simultaneously. The proposed SFL can not only obtain comparably good or better classification performance than using the pixel-wise classification strategy but also takes much less time. In addition, spatial filtering and the non-negative constraints are both adopted to improve the classification performance, and the spatial filtering has a larger effect on the classification than the non-negative constraint. Moreover, methods using $\ell_{2,1}$ norm regularization term can generally obtain better classification performance than methods using an $\ell_{1}$ norm regularization term, which demonstrate that it is beneficial to select correlated training samples among the whole training data set, and the $\ell_{2,1}$ norm regularization term can impose sparsity across the pixels both at the group and individual levels. Furthermore, methods using $\ell_{2,1}$ norm loss function can generally obtain better classification performance than methods using *F* norm loss function, which demonstrate that the $\ell_{2,1}$ norm loss function is more robust for outliers than *F* norm loss function. Finally, experiments on two real hyperspectral image data sets demonstrate that the proposed SFL method outperforms some other popular classifiers. In our future work, we can adopt the CNN framework to extract deep features of hyperspectral images, which can be integrated into our method to improve the classification performance.

## Figures and Tables

**Table 1:** Sixteen ground-truth classes in Aviris Indian Pines and the training and test sets for each class.

Class		Samples	
No.	Name	Train	Test
1	Alfalfa	5	41
2	Corn-notill	143	1285
3	Corn-min	83	747
4	Corn	24	213
5	Grass/Pasture	48	435
6	Grass/Trees	73	657
7	Grass/Pasture-mowed	3	25
8	Hay-windrowed	48	430
9	Oats	2	18
10	Soybeans-notill	97	875
11	Soybeans-min	246	2209
12	Soybeans-clean	59	534
13	Wheat	21	184
14	Woods	127	1138
15	Buildings-Grass-Trees-Drives	39	347
16	Stone-Steel Towers	9	84

**Table 2:** Nine classes in the University of Pavia and the training and test sets for each class.

Class		Samples	
No	Name	Train	Test
1	Asphalt	66	6565
2	Meadows	186	18,463
3	Gravel	21	2078
4	Trees	31	3033
5	Metal sheets	13	1332
6	Bare soil	50	4679
7	Bitumen	13	1317
8	Bricks	37	3645
9	Shadows	9	938

**Table 3:** Overall Accuracy, Average Accuracy, Kappa Statistic and Time of the Indian Pines data set when using pixel-wise strategy.

	Spatial Filtering	No	No	Yes	Yes	No	No	Yes	Yes
Method	Non-Negative Constraint	No	Yes	No	Yes	No	Yes	No	Yes
	Norm	$\ell_{2}$+$\ell_{1}$				$\ell_{1}$+$\ell_{1}$			
	1	14.63	39.02	95.12	100.00	17.07	53.66	95.12	100.00
	2	67.16	73.07	97.90	99.84	68.40	76.19	97.74	99.46
	3	36.68	56.22	92.90	97.32	37.08	58.37	96.39	97.19
	4	20.66	33.33	97.18	97.18	21.13	30.05	96.71	98.12
	5	75.63	90.11	96.78	99.54	76.09	91.49	96.09	99.31
	6	86.15	94.67	95.89	99.39	87.52	96.04	98.63	99.70
	7	4.00	24.00	32.00	84.00	4.00	32.00	36.00	80.00
	8	97.67	100.00	100.00	100.00	97.91	99.77	100.00	100.00
Class	9	5.56	11.11	38.89	55.56	5.56	16.67	33.33	83.33
	10	38.86	29.14	95.09	95.43	38.29	32.80	96.46	96.34
	11	67.22	80.62	97.96	97.69	67.90	81.58	98.28	98.46
	12	49.81	34.46	97.19	97.38	49.06	39.89	97.94	97.75
	13	76.09	81.52	91.30	94.02	76.63	88.04	90.22	95.65
	14	96.84	98.59	99.12	100.00	97.01	98.95	99.38	100.00
	15	39.77	31.41	96.54	96.54	39.48	36.02	98.56	98.85
	16	91.67	92.86	85.71	94.05	91.67	92.86	91.67	92.86
Overall Accuracy (%)		65.63	71.32	96.64	98.06	66.07	73.34	97.44	98.47
Average Accuracy (%)		54.27	60.64	88.10	94.25	54.68	64.02	88.91	96.06
Kappa Statistic		0.603	0.667	0.962	0.978	0.608	0.690	0.971	0.983
Time (s)		5536	5613	4953	4832	30,168	31,843	32,643	32,998

**Table 4:** Overall Accuracy, Average Accuracy, Kappa Statistic and Time of the Indian Pines data set when dealing with all the test pixels simultaneously.

	Spatial Filtering	No	No	Yes	Yes	No	No	Yes	Yes	No	No	Yes	Yes
Method	Non-Negative Constraint	No	Yes	No	Yes	No	Yes	No	Yes	No	Yes	No	Yes
	Norm	*F*+$\ell_{1}$				*F*+$\ell_{2,1}$				$\ell_{2,1}$+$\ell_{2,1}$			
	1	26.83	14.63	95.12	100.00	36.59	31.71	95.12	100.00	36.59	80.49	100.00	100.00
	2	49.34	67.00	98.13	99.84	65.21	63.42	97.82	99.30	69.57	66.54	99.46	99.47
	3	50.87	34.27	95.72	97.32	38.96	44.44	94.78	96.52	44.18	69.75	97.19	97.86
	4	14.08	22.54	97.18	97.18	22.54	21.60	98.12	100.00	27.70	31.46	97.65	97.65
	5	79.77	76.32	97.47	99.54	85.98	83.68	97.01	98.85	85.06	91.26	99.31	99.31
	6	93.00	87.21	97.26	99.39	93.46	94.22	98.02	99.54	95.89	98.48	99.70	99.85
	7	20.00	4.00	52.00	84.00	4.00	16.00	52.00	84.00	8.00	40.00	80.00	92.00
	8	99.07	97.91	100.00	100.00	99.77	99.53	100.00	100.00	100.00	99.53	100.00	100.00
Class	9	5.56	5.56	33.33	55.66	5.56	11.11	83.33	100.00	5.56	22.22	83.33	100.00
	10	28.91	38.74	95.89	95.43	25.14	26.06	96.69	96.91	31.54	49.49	96.34	97.71
	11	78.86	67.81	98.37	97.69	80.81	76.28	98.14	98.78	78.32	83.88	98.46	98.64
	12	31.27	50.19	97.75	97.38	32.21	34.08	97.75	98.69	41.01	63.86	97.75	98.50
	13	74.46	78.80	89.67	94.02	87.50	82.61	90.76	96.20	94.02	98.37	95.65	97.28
	14	98.33	96.49	99.21	100.00	98.24	97.98	98.95	99.91	98.15	98.51	100.00	100.00
	15	25.94	38.90	98.27	96.54	36.31	33.14	97.69	98.56	42.07	44.09	98.85	98.85
	16	92.86	88.10	84.52	94.05	88.10	94.05	84.52	88.10	91.67	96.43	92.86	92.86
Overall Accuracy (%)		65.42	65.67	97.31	98.49	67.96	71.49	97.41	98.59	70.15	77.35	98.48	98.88
Average Accuracy (%)		54.34	54.28	89.37	94.25	56.27	56.87	93.07	97.21	59.33	70.90	96.03	98.14
Kappa Statistic		0.597	0.603	0.970	0.978	0.625	0.616	0.969	0.984	0.653	0.737	0.982	0.987
Time (s)		168	287	188	366	52	253	68	337	488	527	539	551

**Table 5:** Overall Accuracy, Average Accuracy, Kappa Statistic and Time of the Pavia University data set when using pixel-wise strategy.

	Spatial Filtering	No	No	Yes	Yes	No	No	Yes	Yes
Method	Non-Negative Constraint	No	Yes	No	Yes	No	Yes	No	Yes
	Norm	$\ell_{2}$+$\ell_{1}$				$\ell_{1}$+$\ell_{1}$			
	1	58.42	95.31	75.83	90.92	61.42	82.07	84.52	90.94
	2	90.78	95.26	99.93	99.83	92.62	93.45	99.92	99.73
	3	24.21	56.30	71.94	85.13	26.90	59.24	69.44	82.82
	4	83.12	87.27	94.33	92.71	83.98	82.10	93.37	93.31
Class	5	99.77	99.77	100.00	100.00	99.77	99.70	100.00	100.00
	6	37.26	49.43	61.92	86.64	36.96	62.00	65.74	86.06
	7	10.78	4.48	80.64	97.72	9.95	42.90	56.87	95.44
	8	52.18	38.79	59.92	67.85	53.83	39.62	73.85	73.83
	9	62.58	72.49	42.32	83.90	60.98	89.02	49.47	85.39
Overall Accuracy (%)		69.50	79.22	84.63	92.50	71.01	79.39	86.85	92.80
Average Accuracy(%)		57.68	66.11	76.31	89.41	58.49	72.23	77.02	89.72
Kappa Statistic		0.587	0.716	0.792	0.900	0.606	0.723	0.822	0.904
Time (s)		2715	2788	2729	2756	18,218	18,256	18,273	18,286

**Table 6:** Overall Accuracy, Average Accuracy, Kappa Statistic and Time of the Pavia University data set when dealing with all the test pixels simultaneously.

	Spatial Filtering	No	No	Yes	Yes	No	No	Yes	Yes	No	No	Yes	Yes
Method	Non-Negative Constraint	No	Yes	No	Yes	No	Yes	No	Yes	No	Yes	No	Yes
	Norm	*F*+$\ell_{1}$				*F*+$\ell_{2,1}$				$\ell_{2,1}$+$\ell_{2,1}$			
	1	62.13	82.00	81.78	90.25	67.78	81.78	82.38	90.27	67.27	95.31	86.60	89.61
	2	93.65	93.47	99.64	99.71	94.82	93.53	99.86	99.72	96.14	95.26	99.96	99.67
	3	24.11	59.10	68.33	85.61	21.17	59.19	70.12	85.42	39.51	56.30	79.50	88.64
	4	84.70	82.20	93.64	92.68	85.00	82.23	92.91	92.75	86.58	87.27	94.56	93.60
Class	5	99.77	99.70	100.00	100.00	99.70	99.70	100.00	100.00	99.77	99.77	100.00	100.00
	6	37.76	61.98	74.05	88.97	35.87	61.78	71.92	89.05	36.77	49.43	81.34	89.68
	7	3.19	42.52	40.39	97.11	2.43	42.52	43.43	96.74	18.38	0.38	95.52	98.03
	8	57.53	39.78	73.39	70.89	60.52	40.30	74.10	70.86	58.93	38.79	78.74	72.87
	9	43.92	88.70	51.81	83.80	43.50	88.91	55.54	84.54	57.89	72.49	65.03	87.53
Overall Accuracy (%)		71.72	79.50	86.75	92.88	72.57	79.62	87.09	92.90	76.10	81.23	92.29	93.34
Average Accuracy (%)		56.31	72.16	75.89	89.89	56.75	76.70	66.11	89.93	62.36	74.11	86.80	91.07
Kappa Statistic		0.608	0.722	0.821	0.905	0.624	0.723	0.824	0.905	0.656	0.716	0.887	0.911
Time (s)		147	452	169	437	66	433	108	477	611	637	621	648

**Table 7:** Overall Accuracy, Average Accuracy, Kappa Statistic and Time of the Indian Pines data set when using the compared methods and the proposed methods.

Method		SVM	NRS	CRT	KCRC	OMP	SOMP	JRSRC	3D-CNN	SFL
	1	41.46	60.98	26.83	36.59	60.98	68.29	68.29	75.61	100.00
	2	80.39	51.98	87.24	72.84	67.32	94.63	90.97	84.98	99.77
	3	66.93	26.91	55.02	55.15	51.67	86.48	95.31	72.82	97.86
	4	68.54	30.05	27.70	24.41	38.50	89.20	66.67	66.67	97.65
	5	88.51	88.74	91.95	83.22	85.98	95.63	95.17	89.20	99.31
	6	94.67	94.82	99.24	98.17	93.15	99.24	99.39	96.96	99.85
	7	32.00	48.00	20.00	24.00	36.00	12.00	4.00	40.00	92.00
	8	99.53	100.00	100.00	100.00	99.53	100.00	100.00	99.30	100.00
Class	9	33.33	33.33	11.11	22.22	38.89	11.11	11.11	22.22	100.00
	10	74.86	24.91	65.83	56.11	51.54	80.69	71.09	80.23	97.71
	11	84.52	97.87	84.43	90.00	70.08	92.21	98.10	82.16	98.64
	12	84.83	32.58	67.42	44.19	46.07	88.01	96.25	80.90	98.50
	13	99.46	97.28	98.91	98.37	95.65	99.46	100.00	99.46	97.28
	14	97.10	98.33	97.36	98.51	95.08	100.00	100.00	95.08	100.00
	15	43.23	53.31	51.59	40.63	41.50	67.72	65.71	52.74	98.85
	16	94.05	85.71	91.67	90.48	86.90	98.81	100.00	92.86	92.86
Overall Accuracy (%)		82.81	70.74	80.65	76.95	70.57	91.44	92.02	84.04	98.88
Average Accuracy (%)		73.96	64.05	67.27	64.68	66.18	79.52	78.18	76.95	98.14
Kappa Statistic		0.803	0.652	0.777	0.732	0.662	0.902	0.908	0.818	0.987
Time (s)		3	40	147	33	10	145	4231	1895	551

**Table 8:** Overall Accuracy, Average Accuracy, Kappa Statistic and Time of the Pavia University data set when using the compared methods and the proposed methods.

Method		SVM	NRS	CRT	KCRC	OMP	SOMP	JRSRC	3D-CNN	SFL
	1	89.02	92.60	82.96	82.99	67.83	91.96	99.19	89.38	89.61
	2	99.00	98.94	99.26	97.87	93.48	100.00	98.94	99.88	99.67
	3	14.58	58.23	49.37	29.84	55.63	65.78	67.28	88.98	88.64
	4	85.46	85.23	86.38	80.42	79.49	85.00	93.34	93.44	93.60
Class	5	98.35	98.27	99.62	97.75	99.62	100.00	100.00	100.00	100.00
	6	47.96	52.16	55.11	34.61	57.96	63.04	76.64	86.93	89.68
	7	6.45	43.43	61.96	60.06	55.28	86.48	73.58	98.48	98.03
	8	96.84	87.96	87.22	91.91	68.23	71.08	86.26	68.86	72.87
	9	99.68	91.68	90.72	100.00	80.70	80.38	89.23	86.46	87.53
Overall Accuracy (%)		83.27	86.58	85.80	81.89	79.02	88.31	92.34	92.72	93.34
Average Accuracy (%)		70.82	78.71	79.18	75.05	73.14	82.64	87.16	90.27	91.07
Kappa Statistic		0.770	0.817	0.807	0.751	0.719	0.841	0.897	0.903	0.911
Time (s)		3	60	110	56	15	369	1086	663	648
